# Polybrominated Diphenyl Ethers Induce Developmental Neurotoxicity in a Human *in Vitro* Model: Evidence for Endocrine Disruption

**DOI:** 10.1289/ehp.0901435

**Published:** 2009-12-07

**Authors:** Timm Schreiber, Kathrin Gassmann, Christine Götz, Ulrike Hübenthal, Michaela Moors, Guido Krause, Hans F. Merk, Ngoc-Ha Nguyen, Thomas S. Scanlan, Josef Abel, Christine R. Rose, Ellen Fritsche

**Affiliations:** 1 Group of Toxicology, Institut für umweltmedizinische Forschung gGmbH an der Heinrich Heine-Universität, Düsseldorf, Germany; 2 Institute for Neurobiology, Heinrich-Heine-University, Düsseldorf, Germany; 3 Department of Dermatology and Allergology, University clinic, RWTH Aachen University, Aachen, Germany; 4 Departments of Pharmaceutical Chemistry and Cellular and Molecular Pharmacology, University of California–San Francisco, San Francisco, California, USA; 5 Department of Physiology and Pharmacology, Oregon Health and Science University, Portland, Oregon, USA

**Keywords:** brain, development, human, in vitro, neural, neural progenitor cells, neurosphere, PBDE, toxicology

## Abstract

**Background:**

Polybrominated diphenyl ethers (PBDEs) are persistent and bioaccumulative flame retardants, which are found in rising concentrations in human tissues. They are of concern for human health because animal studies have shown that they possess the potential to be developmentally neurotoxic.

**Objective:**

Because there is little knowledge of the effects of PBDEs on human brain cells, we investigated their toxic potential for human neural development *in vitro*. Moreover, we studied the involvement of thyroid hormone (TH) disruption in the effects caused by PBDEs.

**Methods:**

We used the two PBDE congeners BDE-47 and BDE-99 (0.1–10 μM), which are most prominent in human tissues. As a model of neural development, we employed primary fetal human neural progenitor cells (hNPCs), which are cultured as neurospheres and mimic basic processes of brain development *in vitro*: proliferation, migration, and differentiation.

**Results:**

PBDEs do not disturb hNPC proliferation but decrease migration distance of hNPCs. Moreover, they cause a reduction of differentiation into neurons and oligodendrocytes. Simultaneous exposure with the TH receptor (THR) agonist triiodothyronine rescues these effects on migration and differentiation, whereas the THR antagonist NH-3 does not exert an additive effect.

**Conclusion:**

PBDEs disturb development of hNPCs *in vitro* via endocrine disruption of cellular TH signaling at concentrations that might be of relevance for human exposure.

Polybrominated diphenyl ethers (PBDEs) are persistent and bioaccumulative flame retardants that are of concern because they are ubiquitous, are potentially toxic, and have been found at rapidly rising levels in humans during the past few decades [reviewed by Costa et al. (2008)]. PBDEs are widely used by industry as flame retardants in, for example, textiles, electrics, plastics, and furniture. Over time, PBDEs diffuse out of the matrix and bioaccumulate in the environment (Schecter et al. 2006). Furthermore, these chemicals are primarily indoor pollutants and are found at high levels in household dust and other home and workplace environmental samples (Schecter et al. 2005a; Stapleton et al. 2005). The abundance in household dust especially causes high exposure of toddlers and children (Fischer et al. 2006). PBDEs also accumulate in the human body; very high levels have recently been found in milk (Kalantzi et al. 2004; Schecter et al. 2003, 2005b), blood including fetal blood (Mazdai et al. 2003; Morland et al. 2005; Schecter et al. 2005b; Sjodin et al. 2004), placenta (Doucet et al. 2009), and adipose tissue (Johnson-Restrepo et al. 2005; She et al. 2002). Although levels of dioxins, dibenzofurans, and polychlorinated biphenyls (PCBs) have been declining in human tissues, PBDE levels have increased substantially during the past two decades (Schecter et al. 2005b; Sjodin et al. 2004).

The high levels of PBDEs in the human population, especially in infants and toddlers, are of vast concern because these compounds are chemically similar to PCBs and have been shown to be developmentally neurotoxic in rodents. Various PBDE congeners cause behavioral alterations, such as hyperactivity, and disrupt performance in learning and memory tests in perinatally exposed mice and rats [reviewed by Costa et al. (2008)].

Despite extensive information on human exposure and body burden, there is no information on possible neurodevelopmental adverse effects in humans from PBDE exposure. Therefore, any potential risk for adverse nervous system effects in humans has to be extrapolated from animal studies (Costa et al. 2008). To facilitate PBDE hazard assessment for humans, we investigated the impact of PBDEs on human neurodevelopment *in vitro* and studied the mechanisms underlying these changes. For these analyses, we used two of the most prominent congeners found in human tissues, the tetrabrominated congener BDE-47 and the pentabrominated congener BDE-99 [reviewed by Costa et al. (2008)]. We investigated the effects of these PBDEs on the developmental neurotoxicity (DNT)–specific end points proliferation, migration, and differentiation, as well as cell viability, in a human model that mimics brain development *in vitro* (Fritsche et al. 2005; Moors et al. 2007, 2009, 2010). Furthermore, we performed competition studies with the thyroid hormone (TH) receptor (THR) agonist triiodothyronine (T_3_) and the THR antagonist NH-3, or {4-[4-Hydroxy-3-isopropyl-5-(4-nitrophenylethynyl)-benzyl]-3,5-dimethyl}-acetic acid, to investigate the involvement of TH disruption in the observed effects by PBDEs.

## Materials and Methods

### Chemicals

BDE-47 and BDE-99 were a kind gift from U. Strähle from the Karlsruhe Institute of Technology and were diluted in dimethyl sulfoxide (DMSO) at stock concentrations of 1, 10, and 100 mM (purity of both PBDEs were 98.88%). ^14^C-BDE-47 (55 μCi/mg, stated purity > 96%) was a kind gift from K. Crofton from the U.S. Environmental Protection Agency and was diluted in toluene at a stock concentration of 131 mM. T_3_ (Sigma-Aldrich, Munich, Germany) and NH-3 (Nguyen et al. 2002) were diluted in ethanol (300 mM) and DMSO (1 mM), respectively.

### Cell culture

Normal human neural progenitor cells (hNPCs; Lonza Verviers SPRL, Verviers, Belgium) generated from gestational week 16 were cultured as free-floating neurospheres in proliferation medium [Dulbecco’s modified Eagle medium and Hams F12 (3:1) supplemented with B27 (Invitrogen GmBH, Karlsruhe, Germany), 20 ng/mL epidermal growth factor (EGF; Biosource, Karlsruhe, Germany), 20 ng/mL recombinant human fibroblast growth factor (rhFGF; R&D Systems, Wiesbaden-Nordenstadt, Germany), and penicillin and streptomycin (1:100 vol/vol; Invitrogen) at 37°C with 7.5% CO_2_ as previously described (Moors et al. 2007, 2009). Differentiation was initiated by growth factor withdrawal in differentiation medium [Dulbecco’s modified Eagle medium and Hams F12 (3:1) supplemented with N2 (Invitrogen)] and plating onto a poly-d-lysine/laminin matrix.

### Chemical exposure

For viability, migration, and differentiation analyses, neurospheres were preincubated for 1 week with PBDEs (0.1, 1, or 10 μM) in proliferation medium; afterward, differentiation was initiated and spheres differentiated with the same concentrations of PBDEs in differentiation medium for 48 hr (migration measurements) or 7 days (differentiation analyses). This treatment scheme is supposed to imitate exposure of fetal cells during expansion and differentiation *in vivo*. For proliferation analyses, neurospheres were treated for 2 weeks with PBDEs (0.1, 1, or 10 μM) in proliferation medium. For cotreatment with T_3_ or NH-3, spheres were incubated for 48 hr (migration) or 7 days (differentiation) with the indicated concentrations after differentiation was initiated.

### Viability assay

Cell viability was measured using the alamarBlue assay (CellTiter-Blue assay, Promega, Madison, WI, USA), which measures mitochondrial reductase activity, and the CytoTox-ONE assay (Promega), which determines lactate dehydrogenase release, according to the manufacturer’s description. In addition, to determine whether PBDEs cause cell death in differentiating cells in the migration area, cells were incubated for 5 min with 0.1% trypan blue (Sigma-Aldrich). Afterward, dead cells were counted under a light-field microscope (Olympus, Hamburg, Germany).

### Proliferation analyses

For proliferation analyses, spheres were cultured in proliferation medium with or without 20 ng/mL EGF/rhFGF as previously described (Moors et al. 2009). After 0 and 14 days, sphere size was determined by software analyses (MetaMorph, version 7.1; Universal Imaging Corp., West Chester, PA, USA).

### Migration assay

For analyses of hNPC migration, the distance from the edge of the sphere to the farthest migrated cells was measured 48 hr after initiation of differentiation at four defined positions per sphere (Moors et al. 2007).

### Immunocytochemistry

After differentiating for 7 days, the cells were fixed in 2% paraformaldehyde for 30 min and stored in phosphate-buffered saline (PBS) at 4°C until immunostaining was performed. For antibodies against intracellular epitopes, the fixed slides were washed twice for 5 min each in PBS containing 0.1% Triton X-100 (PBS-T). After that, slides were incubated with primary antibodies for 30 min at 37°C in PBS-T containing 10% goat serum. After three additional washes with PBS, the cells were incubated for 30 min with appropriate fluorochrome-labeled secondary antibodies in PBS containing 0.1 μg/mL Hoechst 33258 to label cell nuclei, followed by three washes with PBS for 10 min each. After brief drying, slides were mounted with Vectashield mounting medium (Linaris, Wertheim, Germany). For antibodies against cell-surface epitopes, the same protocol was used with the exception that PBS-T was replaced by PBS in all steps. The primary antibodies were mouse monoclonal IgG anti-β(III)tubulin (1:100; Sigma-Aldrich) and mouse monoclonal IgM anti-O4 (1:50; Millipore, Billerica, MA, USA). The appropriate secondary antibodies were coupled with Alexa Fluor 488 or rhodamine red (1:100; Jackson ImmunoResearch, Dianova GmbH, Hamburg, Germany). For analyses, slides were examined with a fluorescence microscope (Olympus), and photomicrographs were taken at the edge of the sphere with a ColorViewXS digital camera (Olympus). Stained cells were counted manually in relation to the total number of nuclei in the field.

### RNA preparation and reverse-transcriptase polymerase chain reaction

Total RNA was prepared from five differentiated neurospheres either untreated or treated with 10 μM PBDE using the Absolutely RNA Microprep Kit (Stratagene, La Jolla, CA, USA). Real-time reverse-transcriptase polymerase chain reaction (RT-PCR) was performed as previously described (Fritsche et al. 2007). Primer sequences for nestin are CAGCTGGCGCACCTCAAGATG (forward) and AGGGAAGTTGGGCTCAGGACT (reverse). Primer sequences for β-actin are CCCCAGGCACCAGGGCGTGAT (forward) and GGTCATCTTCTCGCGGTTGGCCTTGGGGT (reverse).

### Calcium imaging

We treated neurospheres for 1 week with 10 μM BDE-47 or BDE-99. Subsequently, differentiation was induced under ongoing PBDE exposure. After 24 hr, ratiometric calcium imaging was performed using a wide-field epifluorescence system (TILL Photonics, Martinsried, Germany) attached to an upright microscope (Axioskop; Zeiss, Oberkochen, Germany) with a 40× water-immersion objective (0.8 numerical aperture; Olympus Europe, Hamburg, Germany). Excitation was generated by a monochromator; emission was detected by a CCD camera (imago super-VGA; TILL). Cells were passively loaded by addition of the calcium indicator dye fura-2 acetoxymethyl ester (15 μM; Teflabs, Invitrogen) for 90 min. Fura-2 fluorescence was alternately excited at the isosbestic point (357 nm) and at the calcium-sensitive wavelength (380 nm), and the ratio of fluorescence emission (F_357_/F_380_) in regions of interest positioned around cell somata was calculated. Adenosine triphosphate (ATP) and acetylcholine (ACh) were puff-applied using a Picospritzer II (General Valve/Parker Hanifin, Flein/Heilbronn, Germany) coupled to standard micropipettes with a tip diameter of around 1.5 μm (Hilgenberg, Waldkappel, Germany) placed at a distance of approximately 10–20 μm above the cell layer. Data analysis was performed using TILLVision and IgorPro software (Wavemetrics, Lake Oswego, OR, USA). To determine effects on [Ca^2+^]*_i_*, we used the normalized F_357_/F_380_ ratio for puff application. Any change in the normalized ratios to ≥ 1.2 was considered an increase and was used for further data analysis.

### ^14^C-BDE-47 accumulation

After mitogen withdrawal neurospheres were allowed to attach to culture dish for 4 hr; afterward, cells were exposed to 1 μM ^14^C-BDE-47. The cells were incubated for 7 days at 37°C, and half of the medium was changed every 2 days. At the end of the incubation period, the medium was removed, and cells were washed once with 500 μL PBS. Cells were lysed in 100 μL lysis buffer (Stratagene). ^14^C-BDE-47 concentrations were determined by liquid scintillation counting in residual medium and cell lysates in Roti-Szint (Carl Roth, Karlsruhe, Germany). Intracellular ^14^C-BDE-47 concentrations were calculated after background subtraction (same treatment without spheres) by a standard concentration curve and normalized to sphere volumes. Percent nonspecific binding to the culture dish was determined by subtracting intracellular and medium ^14^C-BDE-47 from total ^14^C-BDE-47 added to the cultures.

### Statistics

For multifactor analyses analysis of variance in combination with the Bonferroni post hoc test was used. Student’s *t*-test was used for two group comparison. The significance value was set at *p* ≤ 0.05.

## Results

### Effects of PBDEs on hNPC viability

To determine viability of cells, we preincubated spheres for 7 days with different concentrations of BDE-47 or BDE-99 under proliferating conditions followed by further differentiation in presence of PBDEs for 7 days or for 14 days as proliferating spheres. Neither mitochondrial activity nor release of lactate dehydrogenase changed significantly compared with the DMSO controls. Additionally, visual inspection of migration areas after staining with 0.1% trypan blue indicated that in all samples the number of dead or damaged cells was < 1% (data not shown). Thus, BDE-47 or BDE-99 did not cause cytotoxicity of hNPC [see Supplemental Material, Figure 1 (doi:10.1289/ehp.0901435)].

### Effects of PBDEs on hNPC proliferation

For assessment of hNPC proliferation, we cultured neurospheres with and without PBDEs (0.1–1 μM) for 2 weeks. We determined increases in cell number by measuring sphere diameter (Moors et al. 2009). In contrast to the negative control without mitogens, PBDEs did not impair sphere growth over time [see Supplemental Material, Figure 2 (doi:10.1289/ehp.0901435)].

### Effects of PBDEs on hNPC migration

**Figure 1:**
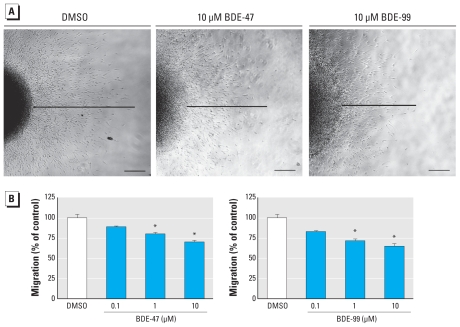
PBDEs inhibit migration of neural progenitor cells: phase-contrast images (*A*) and quantification (*B*) of cell migration. Migration distance was measured at four defined spots from the edge of the sphere to the farthest migrated cell after 48 hr. All data are mean ± SE of three independent experiments (five spheres/experiment). Bars, 200 μm. *p ≤ 0.05.

To measure migration, we determined the distance between the sphere edge and the farthest migrated cells 48 hr after plating. The solvent controls migrated 942.1 ± 42.9 μm within 48 hr, whereas BDE-47–treated hNPCs migrated 838.9 ± 11.6 μm (89.0 ± 1.2% of control; 0.1 μM), 755.3 ± 20.7 μm (80.2 ± 2.2% of control; 1 μM), and 660.7 ± 21.4 μm (70.1 ± 2.3% of control; 10 μM) and BDE-99 exposed cells migrated 781.9 ± 14.8 μm (83.0 ± 1.6% of control; 0.1 μM), 674.5 ± 24.4 μm (71.6 ± 2.6% of control; 1 μM), and 609.6 ± 33.6 μm (64.7 ± 3.6% of control; 10 μM). These data show that PBDEs reduce hNPC migration in a concentration-dependent manner (Figure 1).

### Effects of PBDEs on hNPC differentiation

**Figure 2:**
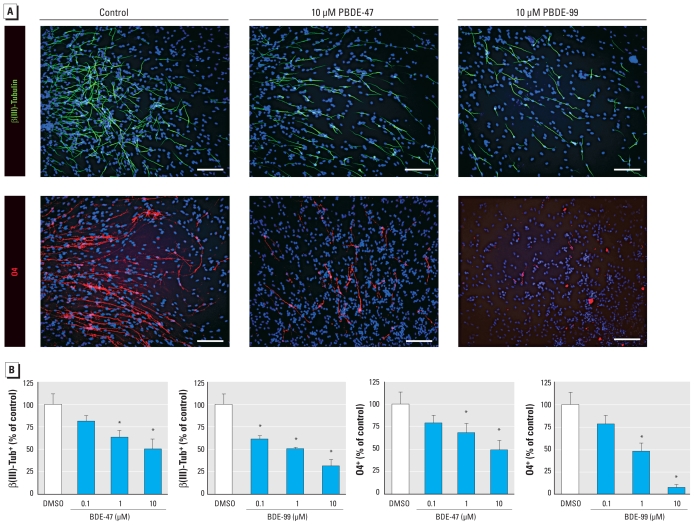
PBDEs inhibit differentiation of hNPCs. (*A*) Representative photomicrographs of hNPCs after 7 days of differentiation. Cells were stained with antibodies against β(III)tubulin [β(III)Tub^+^] for neurons and O4^+^ for oligodendrocytes. Cell nuclei were counterstained with Hoechst. Bars, 50 μm. (*B*) Quantification of immunostaining after PBDE treatment. All data are mean ± SE of three independent experiments (five spheres/experiment). *p ≤ 0.05.

To investigate the influence of PBDEs on differentiation of hNPCs, we preincubated spheres with PBDEs for 7 days under proliferating conditions. After 7 additional days of differentiation under PBDE exposure, we performed immunocytochemical staining for β(III)tubulin (neurons) and O4 (oligodendrocytes; Figure 2). The number of control and PBDE-exposed nuclei in the differentiation zones, visualized with Hoechst, did not differ [see Supplemental Material, Figure 3 (doi:10.1289/ehp.0901435)]. Manual counting of immunopositive cells under blinded conditions revealed that 26.6 ± 3.2% of the control cells were positive for β(III)tubulin, whereas PBDE exposure reduced immunopositive cells to 21.6 ± 1.8% (0.1 μM), 16.8 ± 2.1% (1 μM), and 13.3 ± 3.0% (10 μM) for BDE-47 and to 16.3 ± 1.5% (0.1 μM), 13.4 ± 0.5% (1 μM), and 8.3 ± 2.0% (10 μM) for BDE-99. The effects of PBDE exposure on oligodendrogenesis were stronger than on neurogenesis. Although 5.7 ± 0.8% of all differentiated cells were immunoreactive for O4 after 7 days of differentiation in the control cultures, in the BDE-47–exposed groups only 4.5 ± 0.5% (0.1 μM), 3.9 ± 0.6% (1 μM), and 2.8 ± 0.6% (10 μM) stained O4 positive, and in the BDE-99–treated cells 4.5 ± 0.6% (0.1 μM), 2.7 ± 0.6% (1 μM), and 0.4 ± 0.4% (10 μM). Thus, PBDEs inhibit neural differentiation of hNPCs in a concentration-dependent manner.

**Figure 3:**
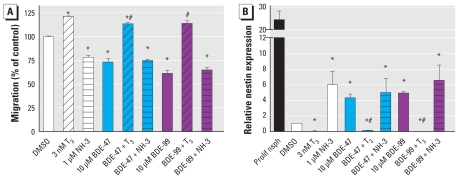
PBDEs disrupt cellular TH signaling. (*A*) hNPC migrated for 48 hr in the presence of the indicated substances, and migration distance was quantified. (*B*) hNPCs differentiated for 7 days in the presence of the indicated substances. Proliferating neurospheres (Prolif nsph) were used as positive control. Real-time PCR analyses for nestin were quantified with a product-specific copy number standard and normalized for β-actin expression. All data (% DMSO control) are shown as mean ± SE of three independent experiments (five spheres/experiment). *p ≤ 0.05 versus control; ^#^*p* ≤ 0.05 versus respective PBDE treatment.

To determine whether PBDEs lead to cell type–specific inhibition of migration, causing cells to remain within the sphere, or whether PBDEs lead to an actual delay in differentiation, we performed real-time RT-PCR analyses of the entire spheres with nestin, a marker of undifferentiated progenitor cells. Control spheres displayed only weak expression of nestin after 7 days of differentiation. In contrast, BDE-47 and BDE-99 increased nestin expression 4- and 5-fold, showing that PBDEs delay differentiation of hNPCs (Figure 3B).

### PBDEs interfere with THR signal transduction

To study involvement of endocrine disruption in the observed PBDE effects, we employed the THR agonist T_3_ and the antagonist NH-3. After initiating differentiation, we exposed the spheres to 10 μM BDE-47 or BDE-99 with or without 3 nM T_3_ or 1 μM NH-3. Stimulation with T_3_ alone increased migration distance significantly to 1181.6 ± 7.4 μm (121.1 ± 0.6% of control) compared with 975.6 ± 7.4 μm in the controls (Figure 3A). In contrast, NH-3 inhibited migration of hNPCs to 735.2 ± 7.2 μm, (78.2 ± 1.8% of controls). Coadministration of BDE-47 or BDE-99 and T_3_ rescued the inhibitory effects of PBDEs completely, the cells migrated over a distance of 1107.4 ± 8.4 μm (113.5 ± 1.2% of control; BDE-47) and 1109.2 ± 18.6 μm (113.8 ± 2.6% of control; BDE-99). In contrast, cotreatment of PBDEs with NH-3 did not have an additive effect, indicating that these substances inhibit migration through an identical mechanism.

To address the question of whether endocrine disruption of the TH system is also responsible for PBDE-induced changes in differentiation, we analyzed nestin expression 7 days after cotreatment with PBDEs and T_3_ or NH-3. T_3_ rescued the PBDE-induced cellular increase in nestin expression, whereas NH-3 showed no additive effects in combination with PBDEs (Figure 3B). These results support the notion that PBDEs delay neural differentiation by interfering with cellular THR signaling.

### PBDEs do not influence calcium signaling

**Figure 4:**
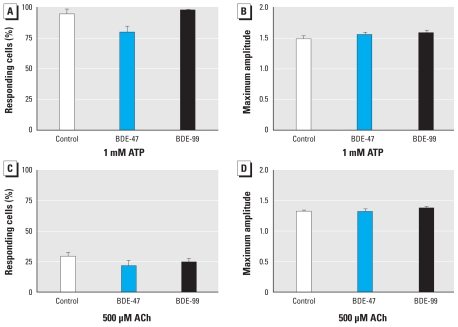
Long-term PBDE exposure does not interfere with calcium signaling. Neurospheres were incubated with 10 μM BDE-47 or BDE-99 for 7 days under proliferating conditions and for an additional day during differentiation. Afterward, hNPCs were loaded with the fura-2 dye and puff-exposed to 1 mM ATP (*A*,*B*) or 500 μM ACh (*C*,*D*). After excitation, the ratio of fluorescence emission (F_357_/F_380_) in regions of interest positioned around cell somata was calculated. Any change in normalized ratios (F_357_/F_380_) ≥ 1.2 was considered as an increase and used for further analysis. All data are mean ± SE of three independent experiments (five spheres/experiment).

We treated neurospheres for 1 week with 10 μM BDE-47 or -99. Subsequently, we induced differentiation under ongoing PBDE exposure. After 24 hr, we stimulated neurospheres with 1 mM ATP or 500 μM ACh to induce calcium signaling. ATP caused a [Ca^2+^]*_i_* increase in 94.7 ± 4.1% of all cells with a maximal amplitude of 1.5 ± 0.1, whereas ACh caused a [Ca^2+^]*_i_* elevation in 29.2 ± 3.6% of all cells with an amplitude of 1.32 ± 0.0. However, BDE-47 and BDE-99 did not change number of responding cells or amplitude of response (Figure 4).

### PBDEs accumulate in hNPCs

Exposure of hNPCs to 1 μM ^14^C-BDE-47 for 7 days resulted in a cellular concentration of 61.16 ± 6.34 μM, which equals an accumulation factor of 60. Only 2% of ^14^C-BDE-47 remained in the media, indicating that 91% of ^14^C-BDE-47 was bound to the culture dish [see Supplemental Material, Figure 4 (doi:10.1289/ehp.0901435)].

## Discussion

Human exposure to brominated flame retardants is of concern because PBDEs impair neurodevelopment in animals, rising concentrations of these compounds are found in human tissues, and nothing is known about their developmentally neurotoxic effects in humans (Costa et al. 2008). Moreover, the mechanisms by which PBDEs interfere with brain development are not known. To shed light onto the effects caused by PBDE exposure in human developing brain cells, we studied their effects on the development of hNPCs *in vitro*. These cells are primary human fetal neuroprogenitors and grow as three-dimensional, complex cellular systems, called neurospheres, in culture. Recently, we have established several end points for DNT testing in such neurospheres—proliferation, migration, differentiation, and apoptosis—by employing end-point–specific controls (Moors et al. 2007, 2009). We showed that this neurosphere system is able to reveal exogenously induced disturbances in these basic processes of brain development. In the present study, we applied this novel testing method to unravel the DNT potential of PBDEs for such developing human cells.

PBDEs (0.1–10 μM) are not cytotoxic for proliferating or differentiating hNPCs over a period of 2 weeks, as we demonstrated by different, independent methods [see Supplemental Material, Figures 1 and 2 (doi:10.1289/ehp.0901435)]. In contrast to our findings, the technical PBDE mixture DE-71 (> 20 μM) caused cell death in rat cerebellar granule cells (Reistad et al. 2006), and BDE-99 (> 25 μM) induced cytotoxicity in human astrocytoma cells (Madia et al. 2004). BDE-47 was reported to cause cell death in hippocampal neurons (41.2 μM), human neuroblastoma cells (> 5 μM), and human fetal liver hematopoietic cells (> 50 μM) (He et al. 2008a, 2008b; Shao et al. 2008). Moreover, the higher brominated congener BDE-209 (> 10 μM) was toxic to human HepG2 hepatoma cells (Hu et al. 2007). The cause of cytotoxicity in all these different cell models was induction of apoptosis. Giordano et al. (2008) demonstrated that DE-71 exposure induces oxidative stress. This production of reactive oxygen species is responsible for DE-71–induced apoptosis of rat cerebellar granule neurons (CGN), as indicated by intracellular glutathione (GSH) content, which is a most important determinant of CGN susceptibility to DE-71 neurotoxicity. Moreover, transgenic rat neuron/astrocyte cocultures with proficient versus deficient GSH synthesis support these findings: Astrocytes rich in GSH protected neurons against DE-71–induced neurotoxicity, whereas astrocytes poor in GSH content did not (Giordano et al. 2009). Protection of neurons against PBDE-induced cytotoxicity by the presence of astrocytes is thus the probable reason for differentiated hNPC insensitivity toward PBDE-dependent cell death, whereas proliferating precursors are known to possess better defense mechanisms than do postmitotic neural cells (Madhavan et al. 2006). Moreover, such human coculture systems, such as differentiated neurospheres, where the three major cell types of the brain are present in “physiological” ratios, seem to be superior *in vitro* methods for assessing hazards of chemicals to humans compared with simple monolayer cell lines.

Neurosphere proliferation assessed by monitoring of sphere diameter (Moors et al. 2009) was also not affected by presence of BDE-47 or -99 [see Supplemental Material, Figure 2 (doi:10.1289/ehp.0901435)]. This is in agreement with results from a T-Screen assay (Gutleb et al. 2005), a functional assay based on T_3_-dependent cell proliferation of the rat GH3 pituitary tumor cell line, where also no effects of these two congeners on proliferation were observed (Hamers et al. 2006). In contrast, one of the hydroxylated BDE-47 metabolites, 2-OH-BDE-47 (5–10 μM), inhibited proliferation of the H295R adrenocortical carcinoma cell line (Song et al. 2009). However, this concentration of reactive metabolite causing an antiproliferative effect in these cells is high, and the researchers did not investigate effects of the parent compound. Inhibition of proliferation by BDE-47 was also seen in 5L rat hepatoma cells (Wahl et al. 2008). However, the researchers demonstrated that arylhydrocarbon receptor (AhR) activation by the BDE-47 contaminant 1,2,3,7,8-pentabromodibenzofuran was responsible for the effects on proliferation, rather than BDE-47 itself. Highly purified BDE-47 does not stimulate the AhR (Peters et al. 2004). This supports our data because PBDEs used in the present study are contaminant-free. Increased proliferation was observed in DE-71–treated MCF-7 breast cancer cells. This stimulation was estrogen receptor dependent, so DE-71 acts as an endocrine disruptor in this estrogen receptor–positive cell line (Mercado-Feliciano and Bigsby 2008). Hence, interaction of PBDEs with cell proliferation seems to be congener and cell type specific. So far, all data available on this topic have been obtained in tumor cells. This is, to our knowledge, the first report employing normal human cells or human stem/progenitor cells for determining effects of PBDEs on cell proliferation.

In contrast to proliferation, both investigated PBDE congeners inhibit migration and differentiation of hNPCs significantly in a concentration-dependent manner (Figures 1 and 2). Consequences of PBDE exposure for cell migration have not been investigated so far in any cell type *in vitro* or *in vivo*, so this is the first report showing that these chemicals have the ability to interfere with human progenitor cell motility. However, a recent proteomics study by Alm et al. (2008) suggests that PBDE exposure might cause disturbances in cell motility also *in vivo*. In that study, a single dose of BDE-99 given on mouse postnatal day 10 caused changes in brain protein expression after 24 hr, and one-third of those proteins were related to the cytoskeleton, including actin. The importance of the actin cytoskeleton for neuronal migration has been reviewed extensively (Luo 2002), leaving room for the speculation that PBDEs might interfere with migration through alteration of cytoskeleton-related protein expression.

Similar to PBDE effects on neural migration, to our knowledge, consequences of PBDE exposure for neural differentiation have not been studied so far. Therefore, this is the first report showing that this group of flame retardants can directly interfere with birth of neurons and oligodendrocytes in this human *in vitro* model. Interference of BDE-47 with neuronal differentiation was also suggested in the fathead minnow because this congener reduced basic transcription element–binding protein (BTEB) expression in their brains (Lema et al. 2008). BTEB is known to be involved in neural differentiation in rodents (Denver et al. 1999). Moreover, BDE-99 altered protein expression of growth-associated protein-43 and brain-derived neurotrophic factor in developing rodent brains (Alm et al. 2008). Both proteins are closely related to neural development and plasticity [reviewed by Strittmatter et al. (1992) and Binder and Scharfman (2004)]. Whether PBDEs disturb brain development by interfering with migration and differentiation not only *in vitro* but also *in vivo* needs to be investigated by appropriate animal experiments. However, because our study was performed in normal cells of human origin, possible species differences should first be addressed by migration and differentiation analyses in rodent neurospheres. Moreover, the developmental stage of cells needs to be considered for rodent *in vitro* and *in vivo* analyses: hNPC generated from gestational week 16 correspond approximately to embryonic day 16 to postnatal day 3 of mouse development.

During normal development, neural migration and maturation of neural and glial cells are guided by TH (Alvarez-Dolado et al. 1999; Wong and Leung 2001). Therefore, hypothyroidism during development causes a large number of neuroanatomical and behavioral effects (Haddow et al. 1999; Schalock et al. 1977; Zoeller and Crofton 2005). Because of similar neurobehavioral alterations observed after PBDE exposure in rodents, endocrine disruption of the TH system by PBDEs has been studied intensively. Hypothyroidism of dams and/or offspring was found in a variety of different studies after pre- or postnatal exposure [reviewed by Costa et al. (2008)]. This reduction in serum thyroxine (T_4_) or T_3_ levels is thought to be caused by induction of the phase II enzyme UDP-glucuronosyl transferase, causing accelerated TH metabolism (Zhou et al. 2002), and by preventing TH from binding to its plasma transport protein transthyretin (Meerts et al. 2000). However, behavioral toxicity of BDE-47 without alterations in serum T_4_ and T_3_ levels was also observed, suggesting that PBDEs cause toxicity by a mechanism beyond changes in body TH homeostasis (Gee and Moser 2008; Gee et al. 2008). These studies might be explained by the data generated in our experiments because the two PBDE congeners BDE-47 and -99 directly disturb migration and delay differentiation of hNPCs *in vitro* by endocrine disruption of cellular TH signaling. We confirmed this by two observations: *a*) PBDE actions are completely antagonized by cotreatment of neurospheres with T_3_ and *b*) simultaneous administration of PBDE and the THR antagonist NH-3 did not cause an additive effect. Although NH-3 binds to THRα as well as THRβ, it has a higher affinity to THRβ (Nguyen et al. 2002). Because inhibition of THRα leads to decreased proliferation of avian neurogenic precursors (Lezoualćh et al. 1995; but PBDEs do not interfere with hNPC proliferation), and the induction of THRβ induces neural differentiation (Jones et al. 2003; Lebel et al. 1994; and PBDEs disturb neural differentiation of hNPCs), it is likely that PBDEs interfere with THRβ signaling of hNPCs. However, reporter gene analyses in THRα and β overexpressing Chinese hamster ovary cells revealed that neither PBDE congener acted as agonist or antagonist of THRα or THRβ. One possible reason for discrepancies between our study and results in the overexpression system might be that PBDEs act via disturbance of recruitment of THR cofactors. In primary cells, receptors, cofactors, and responsive elements are present at a fine-tuned equilibrium, which is not the case in transfected cells, which overexpress only certain elements of the cellular machinery. That nuclear hormone receptor cofactors might be crucial in endocrine disruption by polyhalogenated aromatic compounds is discussed in our previous work (Fritsche et al. 2005), in which we found that the noncoplanar PCB-118 induced oligodendrocyte differentiation in hNPCs. That *in vitro* work represented the finding *in vivo* that Aroclor treatment led to an increased expression of TH-dependent genes such as RC3/neurogranin and myelin basic protein in fetal rat brains (Zoeller et al. 2000). To our knowledge, an equivalent rodent study has not yet been performed with PBDEs. Only in the fathead minnow, TH disruption on the basis of THR-dependent gene expression was observed after BDE-47 exposure (Lema et al. 2008). Thus, this is the first work showing that PBDEs can directly interfere with cellular TH signaling in human neural cells.

Besides endocrine disruption, we also investigated whether PBDEs disturb calcium homeostasis. Calcium signaling is a key player in developmental processes (Ciccolini et al. 2003; Greer and Greenberg 2008), and BDE-47 disturbs calcium homeostasis in rat PC12 pheochromocytoma cells after 20 min of exposure (Dingemans et al. 2008). BDE-47 exerts similar short-term effects on hNPCs (Gassmann K, Krause G, Dingemans M, Schreiber T, Abel J, Bergman A, et al., unpublished observations). To test whether long-term exposure (1 week) to PBDEs also influences calcium signaling and thus contributes to the developmentally neurotoxic effects of PBDEs in hNPC, we measured calcium signaling in PBDE treated hNPCs stimulated with ATP or ACh. Neither BDE-47 nor BDE-99 influenced the response of the cells toward these stimuli, indicating that they do not alter expression of proteins involved in Ca^2+^ flux. Recently, Viberg (2009) showed that BDE-203 and BDE-206 increase Ca^2+^/calmodulin-dependent protein kinase (CaMKII) expression in mouse hippocampus. Whether PBDEs interfere with downstream targets of calcium signaling in hNPCs, such as CaMKII or calcineurin, has to be further elucidated.

In summary, BDE-47 and BDE-99 disturb neural migration and differentiation in a human *in vitro* model for brain development by disruption of cellular TH signaling. The question is now how the lowest observed effect levels from this study relate to actual human PBDE exposure. Therefore, we measured intracellular PBDE concentrations by employing ^14^C-BDE-47. Because medium concentration-dependent intracellular PBDE accumulation *in vitro* follows a linear kinetic (Mundy et al. 2004) and neurosphere material is limited, we measured only 1 μM ^14^C-BDE-47 medium concentration. After 7 days of differentiation, PBDE accumulation is approximately 60-fold. These data support other *in vitro* data where PBDEs accumulate up to 100-fold in neuronal cells (Mundy et al. 2004). It also reflects PBDE accumulation in postnatal day 10 and 19 mouse brains after 7 days *in vivo* (20- to 140-fold) that we calculated from a study of Viberg et al. (2003). PBDE exposure of human infants through breast milk is up to 4,000 ng/kg/day (Jones-Otazo et al. 2005). Assuming an average molecular weight of 500 g/mol for PBDEs, this equals an exposure of 8 nM. Taking into account that there is a 60-fold increase in tissue concentration of PBDEs in our human *in vitro* system and up to a 140-fold increase in brain tissue in mice after oral exposure *in vivo* (Viberg et al. 2003), infant exposure could result in a brain concentration of 0.5–1.1 μM. Considering that 0.1 μM BDE-99 (~ 6 μM tissue concentration) decreases neuronal differentiation by approximately 40% (Figure 2), current PBDE exposure levels are likely to be of concern for human health.

Assessing subtle changes in human IQ or behavior in epidemiologic studies is not trivial and needs large numbers of study subjects. Such investigations are needed to reveal whether PBDEs as a hazard identified in this study actually pose a risk for human brain development *in vivo*.
